# Ramucirumab in combination with dacarbazine in patients with progressive well-differentiated metastatic pancreatic neuroendocrine tumors (RamuNET): study protocol for a multicenter single-arm trial

**DOI:** 10.1186/s12885-021-08900-7

**Published:** 2021-11-12

**Authors:** Sebastian Krug, Thomas Kegel, Thomas M. Gress, Anja Rinke, Leonidas Apostolidis, Henning Jann, Alexander König, Dieter Hörsch, Jörg Schrader, Thomas J. Ettrich, Michael Richter, Jörg Steighardt, Patrick Michl

**Affiliations:** 1 Department of Internal Medicine I, Martin-Luther University Halle/Wittenberg, Ernst-Grube-Str. 40, 06120 Halle (Saale), Germany; 2 Department of Internal Medicine IV, Martin-Luther University Halle/Wittenberg, Halle, Germany; 3 Department of Gastroenterology, Endocrinology and Metabolism, Philipps-University Marburg, Marburg, Germany; 4 Department of Medical Oncology, National Center for Tumor Diseases, University Hospital Heidelberg, Heidelberg, Germany; 5 Department of Gastroenterology and Hepatology, Charité University Hospital, Berlin, Germany; 6 Department of Gastroenterology and gastrointestinal Oncology, Georg-August University, Göttingen, Germany; 7 Department of Gastroenterology/Endocrinology, Center for Neuroendocrine Tumors Bad Berka, Bad Berka, Germany; 8 Department of Medicine - Gastroenterology and Hepatology, University Medical Center Hamburg-Eppendorf, Hamburg, Germany; 9 Department of Internal Medicine I, University of Ulm, Ulm, Germany; 10 Coordination Centre for Clinical Trials, Faculty of Medicine, Martin-Luther University Halle/Wittenberg, Halle, Germany

**Keywords:** PanNET, PNET, Chemotherapy, Ramucirumab, DTIC, Neuroendocrine

## Abstract

**Background:**

Cytotoxic chemotherapy combinations and targeted agents represent established treatment concepts in advanced pancreatic neuroendocrine tumors (PNETs). However, response rates, side effects and outcome data strongly vary among these therapeutic approaches. Head-to-head comparisons between chemo- and molecular therapies are missing and secondary resistances frequently occur. The RamuNET trial aims to identify the effectiveness of dual treatment with DTIC and ramucirumab in progressive advanced PNET patients.

**Methods:**

The RamuNET study is an investigator-initiated multicenter prospective single-arm trial to evaluate the efficacy of ramucirumab in combination with dacarbazine (DTIC) over a period of at least 6 months. Patients with progressive well-differentiated and metastatic pancreatic neuroendocrine tumors are eligible. The study aims to include 45 patients over a period of 24 months with a minimum follow-up of 24 months. The primary endpoint is disease control after 6 months. Secondary endpoints include progression-free survival, biochemical response, overall survival, quality of life and toxicity. Based on the hypothesis that 80% of the patients can achieve a disease control after 6 months, the sample size calculation follows an exact binomial single-stage design. H0: p < =p_0_ = 60% versus H1: p > =p_1_ = 80%, alpha = 0.05, beta = 0.1.

**Discussion:**

This study investigates a new therapeutic approach using the combination of cytotoxic and targeted antiangiogenic therapy in advanced PNET. If positive, this trial will be the basis for a randomized two-arm study to investigate the combination of ramucirumab and DTIC against other established therapies in PNET.

**Trial registration:**

EudraCT: 2017–001207-68. Date of registration: 2018.01.03.

## Background

Worldwide, the incidence of neuroendocrine neoplasms (NEN) has increased over the last decades [1–3]. In contrast to metastatic NEN of the small intestine which are associated with median 10-year survival rates of 60–70%, metastatic pancreatic neuroendocrine tumors (PNET) have a significantly poorer outcome [3, 4]. Numerous efforts have been taken to improve the long-term outcome of PNET patients. However, the impact of current systemic therapeutic approaches is only modest. Recently, two treatment modalities with targeted agents have been approved: The mTOR inhibitor everolimus and the antiangiogenic multikinase inhibitor sunitinib. Both have demonstrated significant clinical efficacy in prolonging PFS in patients with pancreatic NET [5, 6].

Angiogenesis is a key hallmark of neuroendocrine tumors (NET). VEGF signaling has been described as major determinant of the high vascularity seen in NET both in preclinical models and in human disease [7]. Intratumoral and circulating VEGF levels have been associated with increased tumor aggressiveness and reduced survival of NET patients. Several preclinical studies and clinical trials have evaluated the impact of antiangiogenic approaches in patients with pancreatic NET [8]. The anti-angiogenic multikinase inhibitor sunitinib has shown significant effects on PFS as single agent. However, development of secondary resistance is almost inevitable. Likewise, chemotherapy with temozolomide or capecitabine in combination with the anti-VEGF antibody bevacizumab showed moderate improvements of progression-free survival (PFS) in pancreatic NET (PNET) in phase II trials, but secondary resistance is common and phase III data are still missing [9, 10].

The anti-VEGFR2 antibody ramucirumab alone or in combination with chemotherapy has shown significant effects as second-line treatment in gastric cancer patients [11, 12]. In contrast, antiangiogenic strategies using bevacizumab targeting VEGF as ligand have failed [13]. Similar to gastric cancer, pancreatic neuroendocrine neoplasms are characterized by high vascularity and a high stromal content containing various cellular components with high VEGFR2 expression such as macrophages and endothelial cells [14]. Based on the different VEGFR2-targeting mode of action of ramucirumab compared to VEGF-targeting bevacizumab, we hypothesized that ramucirumab is particularly effective in neuroendocrine neoplasms. Besides its efficacy as single agent and in combination with taxane-based chemotherapy in gastric cancer [11, 12], ramucirumab has been approved for non-small cell lung cancer [15] and in combination with FOLFIRI (leucovorin, fluorouracil, irinotecan) for treatment of patients with progressive metastatic colorectal cancer [16].

In NEN, streptozocin-based (STZ) chemotherapy is frequently used and recommended in symptomatic patients with high tumor load [17]. The use of the combination of doxorubicin with STZ is limited by potential cumulative cardiotoxicity (maximum doxorubicin dose must be less than 500 mg/m^2^) and has been largely replaced by the use of the combination of 5-fluorouracil (5-FU) with STZ [18].

However, randomized phase III data are lacking and thus most evidence was achieved with recently published large retrospective studies [19–21]. An alternative therapeutic option is the alkylating drug temozolomide (TEM) or its derivative dacarbazine (DTIC). While TEM is routinely combined with capecitabine, thus presenting an attractive orally available doublet [9], DTIC monotherapy was recently reinvigorated, based on a large retrospective evaluation [22]. Here, DTIC at a dose of 650 mg/m^2^ was used. A 4-week schedule and a favourable toxicity profile represent relevant advantages of DTIC.

## Rationale for the trial

VEGF receptor-2 (VEGFR2) is the premier receptor responsible for many of the cancer phenotypes, including modification of blood vessel structure and function, proliferation and migration [7, 23]. The fully human monoclonal antibody ramucirumab specifically and potently inhibits VEGF receptor-2 [24, 25].

Based on this specificity, ramucirumab has potential advantages over most other antiangiogenic drugs such as bevacizumab as it is selective for VEGFR2, whereas bevacizumab by targeting VEGF-A affects VEGFR1, −R2, and the noncatalytic coreceptors neuropilin-1and − 2. Ramucirumab thus leaves the VEGFR1 receptor which functions as decoy receptor unaffected, thereby further enhancing the VEGFR2 inhibitory effect [24, 25]. Preclinical and clinical data indicate a pivotal role of VEGFR2 in promoting tumor vasculature and progression in PNETs and suggest an impact of VEGFR2 on acquired resistance [14, 26–28].

In addition, VEGFR2 is highly expressed on macrophages, rendering them a relevant target of ramucirumab. Inhibition of VEGFR2 on macrophages results in decreased tumor immune infiltration, cytokine and chemokine release, leading to impaired tumor growth and proliferation [29, 30]. Own preclinical data indicate that macrophages are expressed at high levels in human and murine pancreatic neuroendocrine tumor tissues and are predominantly polarized towards a protumoral, proangiogenic M2 phenotype, therefore representing a promising target for therapeutic intervention.

Combination regimens using a DTIC-based chemotherapeutic backbone combined with ramucirumab may inhibit early therapy-induced resistance mechanisms by targeting angiogenesis synergistically through different mechanisms. Several preclinical or clinical reports demonstrated a marked antiangiogenic action of DTIC or its oral derivative temozolomide indicating synergism with antiangiogenic targeted agents [10, 31].

Moreover, the combination of ramucirumab and DTIC has already been demonstrated as feasible and associated with an acceptable safety profile in a randomized phase II trial in patients with malignant melanoma [10, 31]. Furthermore, ramucirumab was studied in patients with pretreated gastric neuroendocrine carcinomas in combination with chemotherapy. The results were promising and demonstrated a benefit of dual therapy as opposed to chemotherapy alone [32].

Based on the unique molecular mode of action of ramucirumab and the synergistic potential of DTIC, the combination therapy with both agents represents a promising strategy for progressive pancreatic neuroendocrine neoplasms.

## Methods

### Study endpoints

#### Primary endpoints

Primary endpoint is the disease-control (DC) at 6 months as assessed by RECIST 1.1 criteria. Disease-control will be calculated from start of study treatment until progressive disease. DC is the most important parameter to assess the efficacy of a novel therapy regimen in the population of patients with PNET who frequently undergo multiple lines of loco-regional and systemic treatments.

If a positive DC signal with the combination of ramucirumab and DTIC is observed in this pilot trial, a subsequent evaluation in a randomized phase II/III trial is justified.

#### Secondary endpoints

- Objective tumor response (OR)
- progression-free survival (PFS)
- overall survival (OS)
- toxicities
- biochemical response (tumor marker chromogranin A (CgA); in cases of functional pancreatic NET: the specific hormone can be evaluated
- QoL (EORTC QLQ-C30 questionnaire)

### Study population

Patients with histologically confirmed unresectable metastatic non-functional or functional G1-G2 PNET excluding G3 neuroendocrine carcinomas (NEC) or G3 neuroendocrine tumors (NET), who have progressive disease under treatment with either non-DTIC-based chemotherapy (e.g. 5-FU/ streptozocin, capecitabine), SSA analogues, everolimus, sunitinib or PRRT are eligible for screening and enrollment in the trial. The Investigator will keep a record of all study candidates who were considered for enrollment including screening failures.

#### Inclusion criteria

- Histologically confirmed unresectable metastatic non-functional or functional G1-G2 PNET excluding G3 neuroendocrine carcinomas (NEC)
- Age: 18–75 years
- Measurable disease (RECIST 1.1)
- Progressive disease under treatment with either non-DTIC-based chemotherapy (e.g. 5-FU/streptozocin, capecitabine), SSA analogues, everolimus, sunitinib or PRRT. No prior therapy with DTIC is allowed.
- ECOG 0–1
- Life expectancy > 12 weeks
- Adequate renal function (serum creatinine ≤1.5 x ULN, or creatinine clearance (measured via 24-h urine collection) ≥40 mL/minute (if serum creatinine is > 1.5 x ULN, a 24-h urine collection to calculate creatinine clearance must be per-formed). Urinary protein is ≤1+ on dipstick or routine urinalysis (UA; if urine dipstick or routine analysis is ≥2+, a 24-h urine collection for protein must demonstrate < 1000 mg of protein in 24 h to allow participation in this protocol).
- Adequate hepatic function (total bilirubin ≤1.5 mg/dL (25.65 μmol/L), and aspartate transaminase (AST) and alanine transaminase (ALT) ≤ 3.0 x ULN; or 5.0 x ULN in the setting of liver metastases)
- Adequate bone marrow function (absolute neutrophil count > 1500/mm^3^, platelets > 100,000/mm^3^, hemoglobin> 9 g/dL)
- Adequate coagulation function (INR ≤1.5 and PTT ≤ 5 s above the ULN (unless receiving anticoagulation therapy)
- Pathological condition present that carries a high risk of bleeding (for example, tumor involving major vessels or known varices)
- The patient, if sexually active, must be postmenopausal, surgically sterile, or using effective contraception (hormonal or barrier methods, Pearl Index < 1)
- Female patients of childbearing potential must have a negative serum pregnancy test within 7 days prior to first dose of protocol therapy
- The patient must be able to understand, consent and sign the written consent form

#### Exclusion criteria

- Pregnancy (positive urine or blood pregnancy test) or lactation.
- Secondary malignancies in patient’s history with the exception of: disease-free period > 5 years before randomization or basalioma of the skin or carcinoma of the cervix after successful therapy
- Allergy against dacarbacine or ramucirumab
- Current enrolment or participation within the last 4 weeks in a clinical drug trial
- Any arterial thromboembolic events, including but not limited to myocardial infarction, transient ischemic attack, cerebrovascular accident, or unstable angina, within 6 months prior to first dose of protocol therapy
- Insufficient liver function: cirrhosis at a level of Child-Pugh B (or worse) or cirrhosis (any degree) and a history of hepatic encephalopathy or clinically meaningful ascites resulting from cirrhosis. Clinically meaningful ascites is defined as ascites from cirrhosis requiring diuretics or paracentesis.
- Uncontrolled or poorly-controlled hypertension (> 160 mmHg systolic or > 100 mmHg diastolic for > 4 weeks) despite standard medical management
- Chronic antiplatelet therapy, including aspirin, nonsteroidal anti-inflammatory drugs (NSAIDs, including ibuprofen, naproxen, and others), dipyridamole or clopidogrel, or similar agents. Once-daily aspirin use (maximum dose 325 mg/day) is permitted
- Grade 3–4 GI bleeding within 3 months prior to first dose of protocol therapy.
- History of deep vein thrombosis (DVT), pulmonary embolism (PE), or any other significant thromboembolism (venous port or catheter thrombosis or superficial venous thrombosis are not considered “significant”) during the 3 months prior to first dose of protocol therapy
- Uncontrolled severe physical or mental disorders such as: neurological or psychiatric disorders including seizure, advanced dementia, psychosis, active uncontrolled infections or sepsis, HIV, replicative hepatitis B or C infection
- Pathological condition present that carries a high risk of bleeding (for example, tumor involving major vessels or known varices)
- History of gastrointestinal perforation/fistula (within 6 months of first dose of protocol therapy) or risk factors for perforation.
- Major surgery within 28 days prior to first dose of protocol therapy, or minor sur-gery/subcutaneous venous access device placement within 7 days prior to first dose of protocol therapy
- Elective or planned major surgery to be performed during the course of the clinical trial.
- Serious or nonhealing wound, ulcer, or bone fracture within 28 days prior to first dose of protocol therapy.

### Trial design

RamuNET is a multicenter single-arm pilot study. Patients are recruited in approximately 8 participating centres that are all University hospitals or tertiary referral hospitals in Germany with excellent expertise in the care of patients with neuroendocrine neoplasms. A recruitment of an average of 5 patients with progressive PNET per centre over a period of 12 months seems realistic.

### Conduct of the trial

**Fig. 1:**
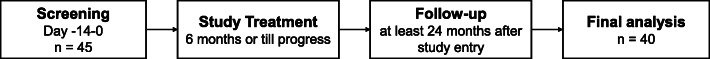
Study procedure

There is a screening period of 28 days. All required study procedures are listed in Table 1. After study enrollment, therapy continues for 6 months (primary endpoint) or until tumor progression. If patients are stable after month 6, treatment can be continued until progression or toxicity). Patients will be followed-up at least 24 months from the start of the study. Finally, the final data analysis is performed (Fig. 1).

**Table 1:**
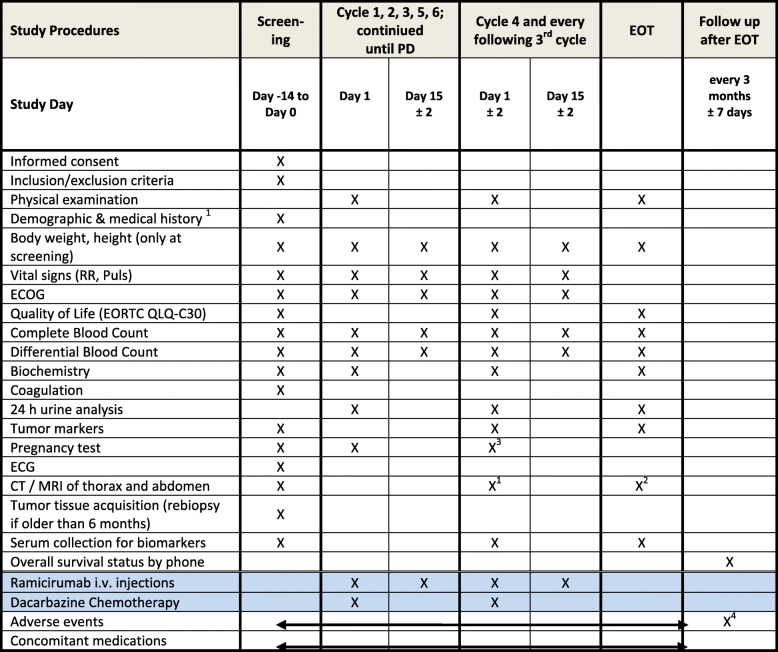
Requirements for the study

^1^every 3 months;

^2^if no imaging has been performed within the last 4 weeks.

^3^to be performed at the beginning of each cycle.

^4^to be followed beyond the 30 days until resolution or stabilization

### Study treatment

**Fig. 2:**
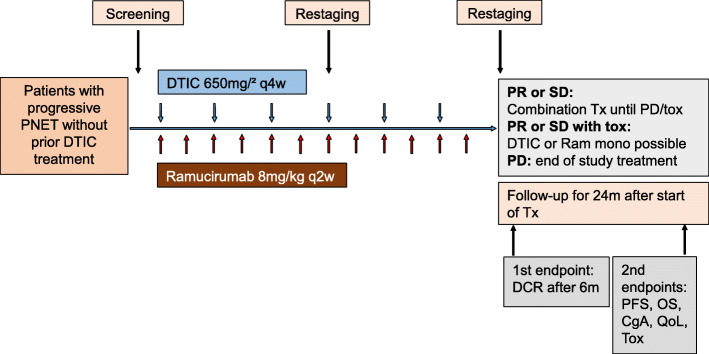
Therapy schedule and endpoints of the study

Ramucirumab at a dose of 8 mg/kg body weight, is administered i.v. on days 1 and 15 of each 28-day cycle, over a time period of 60 min prior to administration of dacarbazine (DTIC). DTIC is given at a dose of 650 mg/m^2^ body surface area on day 1 and then once every 4 weeks as intravenous infusion over 30 min as previously described [22]. Overall the study treatment includes 6 cycles of the combination therapy (Fig. 2).

### Translational program

This study concept is supported by own preclinical data indicating high amounts of VEGFR2 positive tumor-infiltrating macrophages in PNET tissues as therapeutic targets as well as literature reports from other groups and clinical trials in other tumor entities supporting the combination of anti-VEGFR2 together chemotherapy including DTIC. These data indicate that systemic administration of ramucirumab may overcome therapy resistance by affecting both the tumor cell compartment and also the local and systemic innate immune response, leading to a modulation of tumor-associated macrophages towards an antimetastatic phenotype.

The protocol includes analysis of biopsy specimens as well as resected tumor specimens in case of previous surgeries in the participating patients. The aim of the translational program is to correlate response patterns to ramucirumab with characteristic features in the tumor tissue. All participating centers are requested to send tissue blocks from biopsies or previous surgeries for central translational analyses.

Histopathological analysis of tumor specimens will be performed centrally at the Institute of Pathology, Martin Luther University of Halle, Germany. This includes confirmatory analysis of proliferation rate (Ki-67) and tumor grading as well as VEGFR2 expression in tumor and surrounding stromal cells. In addition, infiltration of macrophages (CD68, CD204, CD163, VEGFR2) and different T cell subpopulations (CD3/CD8: cytotoxic T cells, FoxP3: regulatory T cells) will be assessed to correlate infiltration with tumor-associated macrophages and cytotoxic or regulatory T cells with subsequent response to ramucirumab therapy. In addition, immunohistochemical analyses will be performed for angiogenesis (microvessel density; CD31) and epithelial-mesenchymal transition (EMT, E-cadherin, vimentin). Both angiogenesis and EMT are known to be modulated by tumor-associated macrophages and modulate metastasis formation and might serve as predictors of response to bisphosphonates.

In addition to immunohistochemical analyses on resected tumor specimens, we will examine circulating biomarkers in serum to identify predictors of response to ramucirumab or prognostic parameters as liquid biopsies. Serum and plasma will be used for central analysis of circulating, macrophage-associated cytokines and mediators of angiogenesis (e.g. IL1, IL6, VEGF, Angiopoetin-1 / -2) and correlated to clinical parameters.

### Statistical analyses

This trial is planned as a pilot study to evaluate the efficacy of combination treatment of ramucirumab and dacarbazine. Based on the hypothesis that 80% of the patients can achieve a disease control (DCR = CR, PR and SD) after 6 months. The sample size calculation follows an exact binomial single-stage design [33]. H0: p < =p_0_ = 60% versus H1: p > =p_1_ = 80%, alpha = 0.05, beta = 0.1. 45 patients will be recruited in the participating centers during a period of one year. If the primary endpoint of this pilot trial lies within in the confidence interval, thereby demonstrating clinical efficacy of the combination treatment, the aim is to corroborate these data in a randomized phase II study.

### Patient and public involvement

The patient organization Netzwerk Neuroendokrine Tumoren (NeT) e.V. (https://www.netzwerk-net.de; German patient support group for patients with neuroendocrine tumor diseases) was involved and provided precious advice during the planning of the trial and the construction of the trial protocol. Upon trial completion and availability of results, patient involvement will be sought to disseminate the results within the patient community and the public. Furthermore, the present study procotol was presented and discussed in regional and national patient events of the participating centers.

### Ethical considerations

The study will be conducted in compliance with Good Clinical practice (GCP) and the applicable national laws and regulations to assure that the rights, safety, and well-being of the participating subjects are protected consistent with the ethical principles that have their origin in the Declaration of Helsinki. Protocol amendments will be submitted to the ethics committee and signed by all authors of the trial protocol. The Clinical Trials Centre Halle (KKS) is an external party that will monitor the study in a risk-adapted way and ensure that the study follows GCP, that is, that all participants give informed written consent.

### Data management

All data will be captured on paper-based case report forms at the trial site (Case Report Forms – CRF) and transferred to the data management of the KKS Halle. The study management software secuTrial®, a GCP compliant clinical data management system for the conduction of clinical trials will be used for data entry and query management. All changes in the data will be recorded by an audit trail. The study software provides an adaptive concept for user accounts and user roles depending on the study. The data base is integrated in a general IT infrastructure and security concept including a firewall and backup system. The data will be saved on a daily basis. Data will be frequently captured into a data base at KKS Halle. After completion of data capture and quality check the data base will be closed and data will be transferred to the biometrician for statistical analysis.

### Dissemination strategy

Findings of this study will be disseminated to participants, healthcare professionals, the public and other relevant groups. The results of this study will be published open access in a peer-reviewed scientific journal and presented at national and international conferences. The principal investigator will review all manuscripts. The authorship list will be agreed on by the principal investigator prior to publication. Publication of the first manuscript reporting study results is planned after analysis of the primary endpoint.

## Discussion

Chemotherapy with streptozocin (STZ) and 5-FU is considered as first-line treatment in patients with progressive well-differentiated pancreatic neuroendocrine tumor (PNET), although the studies were published a considerable time ago [18, 34]. Recently, somatostatin analogues were shown to prolong the progression-free survival in a subgroup of non-functional PNET patients. After failure of first-line treatment or severe toxicities targeted molecules such as everolimus and sunitinib have been approved after phase III trials demonstrated improvement of PFS. Besides these two trials, randomized therapeutic studies in PNET are rare, given the low prevalence of this disease. Watch and wait strategies in advanced unresectable PNET patients are no longer feasible based on the CLARINET trial demonstrating a benefit for the somatostatin analogue (SSA) lanreotide in PNET. Among all neuroendocrine tumors, the pancreatic tumor site and stage IV disease represent negative prognostic factors. In contrast to low-proliferating NET tumors in other locations for which watch-and-wait strategies can be justified, almost all advanced PNET patients are eligible to receive treatment.

In addition to the fact that randomized evidence on the available therapeutic options is limited, the optimal sequence of available therapeutic options remains also controversial. Since PNET patients frequently undergo multiple lines of treatment and median overall survival (OS) ranges between 5 and 10 years, the influence of one particular therapeutic regimen and its timing in the sequence of therapy lines on OS is moderate hard to assess. Additionally, the combination of different therapeutic protocols e.g. SSA in combination with chemotherapy or targeted agents is not evidence-based but is frequently applied clinical routine. The evaluation of the biologically meaningful combination treatment with the cytotoxic agent dacarbazine (DTIC) and the antiangiogenic molecule ramucirumab represents a first step to generate new evidence in this context and to overcome resistance to currently available treatment options in PNET.

## Trial status

Protocol version 01 was released in August 2018. Recruitment started in June 2019 and the participating centres were successively initiated. Actually, 25 patients have been recruited by all centres. The recruitment period is expected to last another 18 months (status September 2021).

## Data Availability

Not applicable, no datasets are included in this study protocol.

## Abbreviations

CTx: chemotherapy
STZ: streptozocin
TEM: temozolomide
DTIC: dacarbazine
CAP: capecitabine
NEN: neuroendocrine neoplasms
NET: neuroendocrine tumor
w: week
d: day
PFS: progression-free survival
OS: overall survival
RR: response rate
OR: objective response
ORR: objective response rate
DC: disease control
DCR: disease control rate
CR: complete response
PR: partial response
SD: stable disease
PD: progressive disease
ULN: upper level of normal
